# Awake Thoracotomy in an Obese Case of Cardiac Tamponade: A Case Report

**DOI:** 10.1155/cria/7867957

**Published:** 2025-02-11

**Authors:** Malek Itmaiza, Peter Bael, Talha Isayed, Ali Abufarah, Islam Frijat, Majdi Hamamdeh

**Affiliations:** ^1^Medical Research Club, Faculty of Medicine, Al-Quds University, Jerusalem, State of Palestine; ^2^Department of Cardiothoracic Surgery, Al-Ahli Hospital, Hebron, State of Palestine; ^3^Department of Anesthesiology, Al-Ahli Hospital, Hebron, State of Palestine

**Keywords:** awake thoracotomy, case report, obese, parasternal block, serratus anterior block

## Abstract

**Introduction:** Awake thoracic surgery is an old procedure still being used to this day for various indications, utilizing many different techniques for local analgesia.

**Case Presentation:** We described the case of a 60-year-old obese heavy smoker, with significant respiratory and cardiac morbidity, who presented with signs of cardiac tamponade secondary to hypothyroidism. Our patient was treated with a pericardial window, using serratus anterior and parasternal blocks.

**Discussion:** We presented the first case to our knowledge that employed parasternal and serratus anterior blocks as local analgesia for an awake thoracotomy, we also discussed different techniques, indications, benefits, and complications.

## 1. Introduction

The modern era of thoracic surgeries began in the 1950s with the introduction of double-lumen tube ventilation enabling surgeons to operate on a collapsed immobile lung under general anesthesia (GA). This led to the rapid abandonment of the old “Awake Thoracic Surgery,” performed by regional anesthesia techniques in spontaneously breathing, fully conscious patients. Nowadays, it sees occasional use in wedge resections, bullectomies, thymectomy, lung volume reduction surgeries, and the management of pleural effusions to reduce morbidity, hasten recovery, and avoid side effects of GA, especially in high-risk patients [1].

There are several advantages gained from performing awake thoracic surgeries including shorter hospital stays and better patient satisfaction, also considering avoidance of airway trauma associated with endotracheal intubation and single lung ventilation. GA has its risks as it may lead to hyperinflation of the dependent lung, hypoxia due to double lumen endotracheal tube malposition, unilateral ventilator-induced lung injury, and re-expansion pulmonary edema. In addition, avoidance of the use of muscle relaxants can be beneficial in certain patient populations including those with myasthenia gravis [2–5].

Multiple regional anesthesia techniques are implemented throughout the literature including thoracic epidural anesthesia (TEA), intercostal blocks, and rarely paravertebral block, mostly used in conjugation with video-assisted thoracoscopic surgery (VATS), and other thoracoscopic procedures and rarely thoracotomies. In this paper, we presented a case of a 60-year-old man who underwent a pericardial window procedure via awake anterior thoracotomy with parasternal and serratus anterior nerve block as regional anesthesia which to our knowledge has never been recorded in the literature.

## 2. Case Presentation

A 60-year-old morbidly obese male presented to the emergency department complaining of worsening shortness of breath that started 4 days before admission.

He had an extensive history complicated with hypertension, poorly controlled type 2 diabetes mellitus, obstructive sleep apnea, and chronic obstructive pulmonary disorder (COPD).

He was in his usual state of health of diabetic morbidity and limited mobility, until 4 days before admission when he began experiencing progressive shortness of breath and fatigability and increasing facial swelling, prompting him to report to the emergency department. He had a negative Troponin I, and his ECG findings suggested first-degree AV block with occasional ventricular premature complexes, and signs suggesting old anterior myocardial infarction.

At the time, his vitals were consistent type 2 respiratory failure, thus the diagnosis was made. On further examination, his face was puffy and his hands and feet were swollen, consistent with +3 pitting edema of the lower limbs. There was also dullness to percussion on the left lung fields and decreased air entry on the left with left-sided crackles and muffled heart sounds.

He underwent a chest X-ray which showed moderate left-sided pericardial and pleural effusion. He then underwent echocardiography which estimated his ejection fraction at 60%, and a 2.9-cm severe circumferential pericardial effusion, with a swinging heart. Thus, he was transferred to the cardiac surgery department for urgent pericardiocentesis, under the presumption of a cardiac tamponade.

We aspirated the accumulating fluid, a sample of which was sent for cytological evaluation, yielding no significant results.

Follow-up echocardiography revealed mild posterior pericardial effusion.

The patient then underwent full diagnostic work-up including rheumatological and endocrine etiologies. His rheumatological analyses were negative. His thyroid-stimulating hormone was 70 mU/L, and the rest of his thyroid profile was also consistent with hypothyroidism, which was presumed to be the cause of his presentation.

In consultation with the endocrinology team, they determined that the patient was consistent with a picture of severe Hashimoto's disease. He was then started on thyroid hormone replacement therapy, enoxaparin, furosemide, and nifedipine.

One day before his operation, an echocardiographic evaluation revealed a moderate circumferential effusion that was steadily growing. He was hence decided for operative intervention. We initially offered TEA; however, the patient refused and was uncooperative, and with GA being off the table due to the patient's anthropomorphics, difficult airway, and medical history, we opted for local nerve blocks.

Upon patient preparation in the supine position, we provided intravenous sedation with dexmedetomidine (50 μg) and supplemental analgesia with ketamine (25 μg), then parasternal superficial intercostal blockage was carried out under ultrasound guidance. By counting down to the fourth rib, we determined a point 2 cm lateral to the sternum at which we injected 15 mL of 2% lidocaine. Hence, we blocked sensation to the anterior chest wall by targeting the anterior cutaneous branches of the intercostal nerves.

For deep serratus anterior blockage, we used bupivacaine 0.5% 10 mL and 30 mL of 1% lidocaine, and 4 mg dexamethasone lateral to the nipple in the mid axillary line, at the level of the fifth rib neutralizing sensation to the axilla and lateral chest wall by targeting the lateral cutaneous branches of the intercostal nerves. Each region was assessed for pain sensation at the end of administration.

Thus, upon application of locoregional anesthesia, the patient was awake and ready for the operation. He underwent a left anterior thoracotomy, where the left pleura was opened revealing a large effusion of 400 cc, which was aspirated. Then, the pericardium was opened and a large pericardial window was formed. The pericardial effusion was evacuated, and a left pleural chest tube was positioned. The opened layers were then closed, and the operation concluded. Throughout the procedure, and while being transferred to the recovery room, the patient was conscious, coherent, and vitally stable. He was followed up by our team in the recovery room and ward along with a 10-point Numerical Pain Score. The patient's pain was consistently at three during the postoperative period up to 6 h, which gradually dropped to one by the 24-h mark. Notably, the patient's pain increased with ambulation and cough with its worst during the first 6 h being six and its lowest being two after the first day. His first independent ambulation was at 12 h.

Postoperative analgesia was provided using only IV acetaminophen 500 mg four times a day to keep the NRS score less than 3/10. We encountered no complications or adverse effects throughout the postoperative period.

On postoperative echocardiography, there was trace circumferential pericardial effusion, but the patient was well and stable. His respiratory function was adequate, at 88% at room oxygen similar to his baseline. He was discharged on the third postoperative day, with oral analgesia upon need. On follow-up, the patient had no complaints and was doing well.

## 3. Discussion

As mentioned beforehand, surgeries without GA were the standard procedure throughout history but are still used to this date for various indications, utilizing various techniques for regional anesthesia, most commonly TEA. In the aforementioned case, the parasternal and serratus anterior block was used, which to our knowledge of the current literature has never been used as the sole locoregional anesthesia technique for any surgery. Our case aims to shine the light on underused techniques that are easier and safer to perform and might have the same effectiveness as the more commonly used methods.

Thoracic surgeries operated under GA carry multiple risks, most notably the risk of the GA alone, but in most patients, these risks are outweighed by several factors including the most obvious which is the ability to work in the thoracic cavity with a deflated lung, but some patients such as ours are at high risk for GA having newly discovered severe Hashimoto disease, hypertension, poorly controlled type 2 diabetes mellitus and COPD, requiring urgent surgical intervention, and going for surgery under local anesthesia on a spontaneously breathing patient is the only viable option.

There are numerous indications for performing surgeries in awake patients under regional anesthesia in the literature, including high-risk patients who have multiple comorbidities especially COPD, as respiratory status deterioration following GA [6] and ventilator dependency [7]are of major concern, patients who refuse GA and prefer to be awake [2–4, 7], patients with specific indications as mentioned by Chia-Rong Yen [3] who reported a 64-year-old female with polymyositis and bronchiolitis obliterans organizing pneumonia (BOOP) who had severe dyspnea caused by huge pulmonary bulla as surgery under GA with one-lung ventilation would impose serious risk, and Chang Y Park [2] who reported a case of metastatic breast cancer who had an urgent need for chemotherapy, thus a minimally invasive approach coupled with local anesthesia was performed for the quickest recovery time possible, and in some instances, the indication was not specified by the author [5].

Considering the history and applicability of awake surgeries, many methods have been employed. The literature has displayed a tendency for TEA to be the go-to candidate in more invasive procedures, while it was a toss-up for more conservative procedures between TEA and intercostal blocks. Other methods include local wound infiltration, serratus anterior plane block, and lidocaine administration in the pleural cavity. We will only be further discussing TEA, intervertebral block serratus anterior plane block, and intercostal block [1, 8].

Though serratus anterior block has been described for use in multiple rib fractures, it has showed promise in combination with other forms of the block to achieve superior results, even in difficult cases such as obese body habitus, as seen in our patient.

Pompeo has argued that the seldom-used paravertebral block or intercostal block would be a safer option than the more commonly used TEA. Epidural anesthesia has displayed great efficacy in cardiac operations, largely due to its association with decreased oxygenation demand, enhanced ventricular function, improved thrombotic outcomes, and antiarrhythmic and antiischemic effects. It has also showed benefits in operative respiratory function. Inversely, the intercostal block has proven to be safer in anticoagulated patients, as well as those with spinal deformities impeding TEA. The intercostal block also spares patients with the accompanying nausea and hypotension associated with TEA. Gabor et al. have noted that TES and paravertebral block have similar efficacy and pain relief levels, though the paravertebral block comes with an extensively better safety margin, specifically in the case of parasympathetic side effects and other complications. One potential complication is pneumothorax, which is dealt with by simple planned chest tube placement [1, 8, 9].

Despite the thoroughly studied benefits of TEA, it still offers major significant risks. Direct nerve trauma is perhaps most significant when concerning TEA, occurring in up to 1.3% of patients. Further complications include postdural headaches and phrenic nerve palsy.

Another issue is that despite the great lengths of characterization of benefits and risks in TEA for awake thoracic surgeries, the literature is incomplete regarding its competitors, especially considering the benefits may not be mutually exclusive, while the determinants are [1].

## 4. Conclusion

Despite multiple methods of awake anesthesia described in the literature, we emphasize further study into alternative techniques that might offer equal efficacy profiles with decreased side effects such as parasternal block and serratus anterior block.

## Data Availability

The data that support the findings of this study are available on request from the corresponding author. The data are not publicly available due to privacy or ethical restrictions.
